# Supplementary material for: Level of Knowledge Regarding Mpox among Peruvian Physicians during the 2022 Outbreak: A Cross-Sectional Study
Source: Vaccines (Basel). 2023 Jan 12;11(1):167. doi: 10.3390/vaccines11010167 (PMC9860588; doi:10.3390/vaccines11010167)
Supplement: Supplementary file 1 [file vaccines-11-00167-s001.zip › vaccines-2114689-supplementary.pdf]

Supplementary File 1

STROBE Statement—Checklist of items that should be included in reports of cross-sectional studies

	Item No	Recommendation	Page No
Title and abstract	1	(*a*) Indicate the study's design with a commonly used term in the title or the abstract	1
		(*b*) Provide in the abstract an informative and balanced summary of what was done and what was found	1-2
**Introduction**			
Background/rationale	2	Explain the scientific background and rationale for the investigation being reported	2
Objectives	3	State specific objectives, including any prespecified hypotheses	3-4
**Methods**			
Study design	4	Present key elements of study design early in the paper	4
Setting	5	Describe the setting, locations, and relevant dates, including periods of recruitment, exposure, follow-up, and data collection	4
Participants	6	(*a*) Give the eligibility criteria, and the sources and methods of selection of participants	4
Variables	7	Clearly define all outcomes, exposures, predictors, potential confounders, and effect modifiers. Give diagnostic criteria, if applicable	5
Data sources/ measurement	8*	For each variable of interest, give sources of data and details of methods of assessment (measurement). Describe comparability of assessment methods if there is more than one group	*5*
Bias	9	Describe any efforts to address potential sources of bias	NR
Study size	10	Explain how the study size was arrived at	4
Quantitative variables	11	Explain how quantitative variables were handled in the analyses. If applicable, describe which groupings were chosen and why	5
Statistical methods	12	(*a*) Describe all statistical methods, including those used to control for confounding	6
		(*b*) Describe any methods used to examine subgroups and interactions	NR
		(*c*) Explain how missing data were addressed	6
		(*d*) If applicable, describe analytical methods taking account of sampling strategy	4
		(*e*) Describe any sensitivity analyses	NR
**Results**			
Participants	13*	(a) Report numbers of individuals at each stage of study—eg numbers potentially eligible, examined for eligibility, confirmed eligible, included in the study, completing follow-up, and analysed	7
		(b) Give reasons for non-participation at each stage	NA
		(c) Consider use of a flow diagram	7
Descriptive data	14*	(a) Give characteristics of study participants (eg demographic, clinical, social) and information on exposures and potential confounders	7
		(b) Indicate number of participants with missing data for each variable of interest	NR
Outcome data	15*	Report numbers of outcome events or summary measures	8
Main results	16	(*a*) Give unadjusted estimates and, if applicable, confounder-adjusted estimates and their precision (eg, 95% confidence interval). Make clear which confounders were adjusted for and why they were included	9-11

		(*b*) Report category boundaries when continuous variables were categorized	NA
		(*c*) If relevant, consider translating estimates of relative risk into absolute risk for a meaningful time period	NR
Other analyses	17	Report other analyses done—eg analyses of subgroups and interactions, and sensitivity analyses	NR
**Discussion**			
Key results	18	Summarise key results with reference to study objectives	11-14
Limitations	19	Discuss limitations of the study, taking into account sources of potential bias or imprecision. Discuss both direction and magnitude of any potential bias	14-15
Interpretation	20	Give a cautious overall interpretation of results considering objectives, limitations, multiplicity of analyses, results from similar studies, and other relevant evidence	11-14
Generalisability	21	Discuss the generalisability (external validity) of the study results	15
**Other information**			
Funding	22	Give the source of funding and the role of the funders for the present study and, if applicable, for the original study on which the present article is based	15

NR: Not reported, NA: Not applicable. *Give information separately for exposed and unexposed groups. Note: An Explanation and Elaboration article discusses each checklist item and gives methodological background and published examples of transparent reporting. The STROBE checklist is best used in conjunction with this article (freely available on the Web sites of PLoS Medicine at http://www.plosmedicine.org/, Annals of Internal Medicine at http://www.annals.org/, and Epidemiology at http://www.epidem.com/). Information on the STROBE Initiative is available at www.strobe-statement.org.

Supplementary File 2. Survey to assess knowledge of Monkeypox.

Inclusion Criteria

1. Are you a licensed physician in Peru?
   a. No (survey ends)
   b. Yes

2. Are you Peruvian?
   a. No (survey ends)
   b. Yes

3. Do you currently live in Peru?
   a. No (survey ends)
   b. Yes

4. Are you currently practicing Medicine? (Clinical, research, or administrative)
   a. No (survey ends)
   b. Yes

Basic Participant Information

(1) Indicate your gender:
   a. Male
   b. Female

(2) What is your age? (years) ....

(3) Indicate your place of residence:

1. Lima
2. Callao
3. Arequipa
4. Loreto
5. Ica
6. La Libertad
7. Ancash
8. Junín
9. Cajamarca
10. Apurimac
11. Ayacucho
12. Cusco
13. Huancavelica
14. Huánuco
15. Lambayeque
16. Madre de Dios
17. Moquegua
18. Pasco
19. Piura
20. Puno
21. San Martin
22. Tacna
23. Tumbes
24. Ucayali
25. Amazonas

(4) Which university did you complete your undergraduate studies in?

a. Public University
b. Private University

(5) How long ago did you graduate from medical school? (In years)

......

(6) Indicate the medical position you hold:

a. General Physician
b. Resident Physician (in training)
c. Medical Specialist

(7) Do you have any of the following academic degrees?

a. Master's degree
b. PhD
c. Both (a and b)
d. None

(8) What is your specialty in Medicine?

a. I do not have a specialty
b. Internal Medicine
c. Emergencies and Disasters
d. Family Medicine
e. Infectious Diseases
f. Intensive Care
g. Pneumology
h. General Surgery or another surgical specialty
i. Obstetrics/Gynecology

j. Pediatrics
k. another specialty. Specify..........

(9) Which sector do you work in?

a. Public
b. Private
c. Both

(10) Which facility do you work in? (You can choose more than one alternative)

a. Health Post
b. Health Center
c. Polyclinic
d. Public Hospital
e. Private Clinic
f. Research Center
g. University
h. Other. Specify.........

(11) Where is your workplace located?

a. Urban area
b. Rural area

(12) Have you heard about the current outbreak of monkeypox?

a. No
b. Yes

(13) What sources have you used to obtain information about monkeypox (you can choose more than one alternative)?

a. Books or scientific journals
b. Conferences
c. Training courses
d. Official websites of health institutions: MINSA, CDC, WHO, etc.
e. Conversations with colleagues
f. Radio or Television
g. Newspapers or magazines for public
h. Social Networks (Facebook, Instagram, Twitter, TikTok)
i. WhatsApp chains
j. I have not received any information

(14) Have you ever taken care of a patient with a confirmed or probable diagnosis of monkeypox?

a. No
b. Yes

Knowledge questions

1. Monkeypox is an endemic disease of Central and West Africa.

   a. False
   b. True

2. . Cases of monkeypox have been reported in Peru.

   a. False
   b. True

3. Monkeypox is caused by:

a. Bacterium
b. Fungus
c. Virus
d. Parasite

4. Monkeypox can be transmitted to a person by the bite of an infected rodent

a. False
b. True

5. Monkeypox can be transmitted from person to person.

a. False
b. True

6. All are possible routes of transmission of monkeypox, except

a. Direct contact with skin lesions
b. Prolonged and close contact with respiratory secretions
c. Through clothing or linens that were in contact with lesions
d. Mosquito bite

7. According to current data, what is the population most affected by monkeypox in this outbreak?

a. Children 5 to 10 years old
b. Pregnant women
c. Men who have sex with men
d. General heterosexual population

8. . What is the incubation period of monkeypox?

a. 30 to 60 days
b. 5 to 21 days
c. 1 to 4 days
d. 2 to 3 months

9. Monkeypox may present with influenza-like symptoms in the first days of illness.

a. False
b. True

10. Vesicles on the skin are one of the classic lesions of monkeypox.

a. False
b. True

11. Proctitis (inflammation of the rectum) is one of the manifestations of monkeypox.

a. False
b. True

12. Monkeypox lesions do NOT occur on the genitalia.

a. False
b. True

13.. Which test is used to confirm the diagnosis of monkeypox?

a. Serology (antibody titer)
b. PCR testing of lesions
c. Sputum culture
d. Stool examination

14. Persons with symptoms of monkeypox require isolation to limit the spread of the infection.

a. False
b. True

15.. What treatment would you use for people with mild monkeypox symptoms?

a. Ivermectin
b. Azithromycin
c. Acyclovir
d. Symptomatic treatment

16. There is an FDA-approved vaccine for the prevention of monkeypox

a. False
b. True

17. Monkeypox has a mortality rate of approximately 80%.

a. False
b. True

- Thank you very much for your participation.
